# ODMedit: uniform semantic annotation for data integration in medicine based on a public metadata repository

**DOI:** 10.1186/s12874-016-0164-9

**Published:** 2016-06-01

**Authors:** Martin Dugas, Alexandra Meidt, Philipp Neuhaus, Michael Storck, Julian Varghese

**Affiliations:** Institute of Medical Informatics, University of Münster, Albert-Schweitzer-Campus 1 | A11, D-48149 Münster, Germany; European Research Center for Information Systems (ERCIS), Münster, Germany

**Keywords:** Semantic annotation, Personalised medicine, Data integration, ODM

## Abstract

**Background:**

The volume and complexity of patient data – especially in personalised medicine – is steadily increasing, both regarding clinical data and genomic profiles: Typically more than 1,000 items (e.g., laboratory values, vital signs, diagnostic tests etc.) are collected per patient in clinical trials. In oncology hundreds of mutations can potentially be detected for each patient by genomic profiling. Therefore data integration from multiple sources constitutes a key challenge for medical research and healthcare.

**Methods:**

Semantic annotation of data elements can facilitate to identify matching data elements in different sources and thereby supports data integration. Millions of different annotations are required due to the semantic richness of patient data. These annotations should be uniform, i.e., two matching data elements shall contain the same annotations. However, large terminologies like SNOMED CT or UMLS don’t provide uniform coding. It is proposed to develop semantic annotations of medical data elements based on a large-scale public metadata repository. To achieve uniform codes, semantic annotations shall be re-used if a matching data element is available in the metadata repository.

**Results:**

A web-based tool called ODMedit (https://odmeditor.uni-muenster.de/) was developed to create data models with uniform semantic annotations. It contains ~800,000 terms with semantic annotations which were derived from ~5,800 models from the portal of medical data models (MDM). The tool was successfully applied to manually annotate 22 forms with 292 data items from CDISC and to update 1,495 data models of the MDM portal.

**Conclusion:**

Uniform manual semantic annotation of data models is feasible in principle, but requires a large-scale collaborative effort due to the semantic richness of patient data. A web-based tool for these annotations is available, which is linked to a public metadata repository.

## Background

**Fig. 1:**
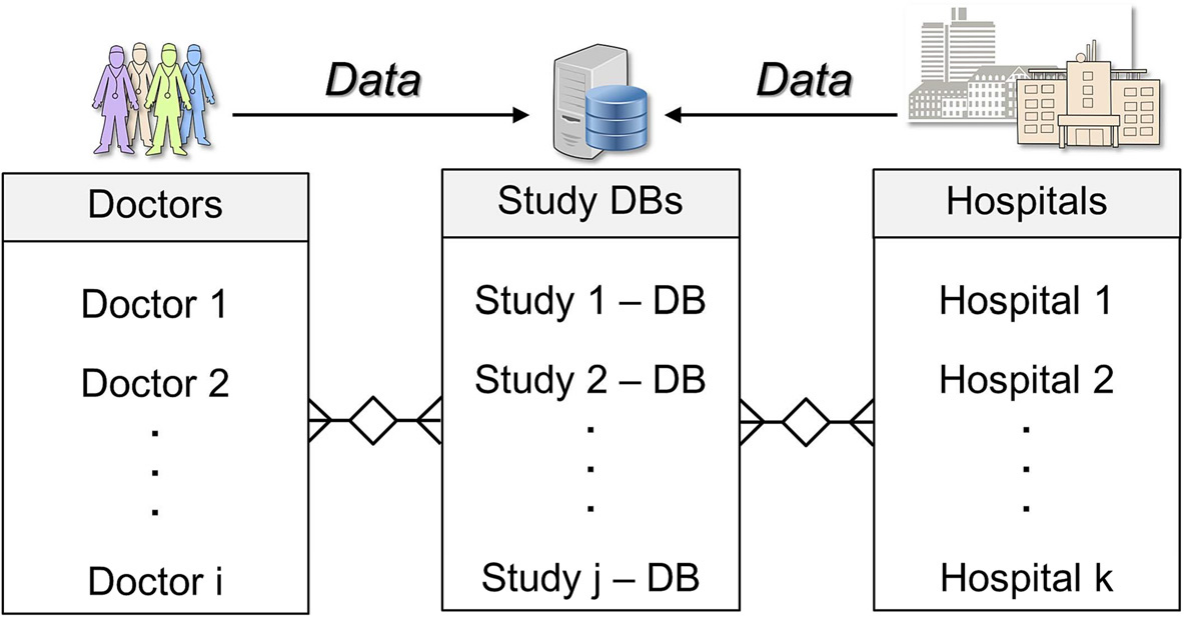
Data flow for clinical studies. Patient data is collected both at doctor’s offices and in hospitals and needs to be transferred to a dedicated study database (DB) for each study. Each study has a unique set of participating doctors and hospitals, therefore many different types of computer systems need to be connected

According to the U.S. National Human Genome Research Institute, personalised medicine “is an emerging practice of medicine that uses an individual’s genetic profile to guide decisions made in regard to the prevention, diagnosis, and treatment of disease” [1]. However, there are very many different profiles, for example regarding cancer [2]. This leads to a very small number of patients with a certain profile. Therefore many clinical sites need to be involved for a clinical study in personalised medicine. Integration of patient data – e.g., non-genomic diagnostics – from multiple clinical sites is a non-trivial problem. Traditional clinical trials collect a large amount of data items [3] – on average 180 pages per patient. Observational studies apply case report forms (CRFs) or re-use routine care data to collect patient data from multiple sites. There is a strong need to exchange patient data from different sources for clinical research. Patient data are nowadays stored in Electronic Health Record (EHR) systems. Figure 1 depicts the general data flow for clinical studies. Each hospital and each practising doctor constitutes a data source, which contributes to a study database.

There is a large variety of EHR systems [4], among other reasons because EHR data are typically collected in the local language of each country and because there are many specialised systems for certain disease domains. These heterogeneous systems, combined with the high number of data items per study, pose significant challenges for data integration. For valid results it is required that the meaning of data is not altered by the data exchange mechanism. In technical terms, this property is called semantic interoperability, which is “the ability of computer systems to exchange data with unambiguous, shared meaning” [5]. There are two major issues addressed in this work regarding semantic interoperability of patient data:

Firstly, data items are not semantically annotated in most current patient data systems, i.e., the precise meaning of data items is not well defined, which jeopardises patient data integration from different sources [6]. Item names can be ambiguous and are therefore inappropriate to capture the meaning of a data item. For example an item named “size” can refer to tumour size in one data source and body height in another. Semantic annotations (i.e., semantic codes associated with data elements, also called terminology bindings) enable ontology-based data integration: International terminologies like Systematized Nomenclature of Medicine Clinical Terms (SNOMED CT) [7] or a metathesaurus like the Unified Medical Language System (UMLS) [8] can help to specify the precise meaning of each data item, for instance UMLS code C0475440 corresponds to tumour size while C0005890 specifies body height. Data integration involves a step of annotating each data item with an appropriate terminology code [6]. Data items, which are represented by the same medical concept, should receive the same terminology code. The assignment of medical terms or data item names to medical concepts should be defined by domain experts. This is challenging given the huge number of medical terms and related homonyms as well as synonyms.

Secondly, the vast majority of medical data structures (i.e., structural metadata) are currently not available to the scientific community, in particular medical forms and their data items [9]. Only eligibility criteria of clinical trials are available on the Internet, corresponding to approximately 1 % of CRFs (1–2 pages of 180 pages on average), i.e., 99 % of CRFs are not public. This holds true both for clinical trials and routine care systems. At present, there is no regulatory requirement for transparency of data structures in medical information systems. Most computer systems in healthcare are commercial and their data structures are not public. However, it is not possible to build interfaces between systems with secret data structures. Data integration in medicine is severely impeded by this issue of intransparency.

Semantic annotation is a key step in data integration, because it enables to identify matching data elements in different data sources. The objective of this work is to demonstrate the feasibility of uniform manual (i.e., expert based) semantic annotation of patient data items based on a public metadata repository.

## Methods

### Technical representation of patient data elements

Data elements are represented according to the ISO/IEC 11179 Standard [10], i.e., both concept domain and value domain (set of permissible values) are specified.

New medical treatments must be assessed in the existing regulatory framework for clinical research. The regulatory agencies – in particular Food and Drug Administration (FDA) and European Medicines Agency (EMA) – accept data from validated Electronic Data Capture (EDC) systems, which predominantly apply standards from the Clinical Data Interchange Standards Consortium (CDISC). In particular, any kind of patient data items can be represented by CDISC Operational Data Model (ODM) [11], an open XML-based transport format standard for both metadata and patient data in clinical trials. CDISC ODM (Define XML) is part of FDA’s Data Standards Catalog, which was announced to become mandatory for new drug applications by end of 2016 [12].

In this context metadata refers to the definition of items – for example “age” as item name, “integer” as data type and “years” as unit –, and patient data to the actual item values, for instance “50”. ODM enables semantic annotation [13] for concept domain and value domain. Converters for several other data formats are available [14]. Therefore ODM was selected as technical representation for patient data items with semantic annotation.

### Open-access metadata repository

Identifiable patient data must be kept private, but metadata like medical forms and its data items need to be publicly available to design interfaces between the multitude of different data sources. Given the large number of patient data sources, open-access to metadata is likely the most efficient solution to foster standardisation and setup of interfaces for patient data integration. The portal of medical data models (MDM) is an open-access repository for metadata in CDISC ODM format [15] with ~5,800 forms and ~450,000 data elements (as of April 2016). At present, it is the largest public portal of medical data models, but still has limited coverage in the context of ~210,000 registered clinical trials [16]. This metadata repository is used for a reference implementation of a novel annotation approach. In particular, it stores semantic annotations for data elements.

### ODMedit: uniform semantic annotation of patient data items

Each data item with the same meaning should be assigned the same terminology code to foster data integration between different patient data sources. Basically, these codes support the decision whether two data items from different systems can be merged or not. Such unique codes are provided by classifications, for example the International Classification of Diseases (ICD) [17]. However, ICD version 10 with its approximately 13,000 codes does not provide the level of detail which is needed to capture the semantic richness of patient data. SNOMED and UMLS (contains >4 million terms) provide much more detail, but don’t provide uniform coding. For example, “patient sex” and “gender of patient” have a very similar human-readable meaning, but different UMLS codes (C0150831 [Organism Attribute T032] and C1548569 [Intellectual Product T170]). Another example: “antidementia drug” (C1276997 [Pharmacologic Substance T121]) and “antidementia agents” (C1531592 [Pharmacologic Substance T121]) can be considered synonyms, but have different UMLS codes. For instance, gastroenteritis with MRSA can be coded in several ways with SNOMED CT (the following list is probably incomplete): A) Staphylococcus Aureus Gastroenteritis (SNOMED CT 32527003), B) MRSA - Methicillin resistant Staphylococcus aureus infection (SNOMED CT 266096002), C) Staphylococcus Aureus Gastroenteritis (SNOMED CT 32527003) + Methicillin resistant Staphylococcus aureus (organism) (SNOMED CT 115329001), D) MRSA - Methicillin resistant Staphylococcus aureus infection (SNOMED CT 266096002) + Gastrointestinal system (SNOMED CT 86762007) or E) Gastroenteritis (SNOMED CT 25374005) + Methicillin resistant Staphylococcus aureus (organism) (SNOMED CT 115329001). SNOMED‐CT has a sub‐type hierarchy, supported by defining relationships based on description logic. There are multiple ways for coding and no formal guidance is available, which SNOMED CT or UMLS coding shall be used.

**Fig. 2:**
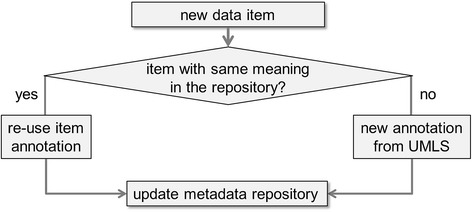
Workflow of ODMedit. To achieve uniform semantic annotations, codes from a metadata repository are re-used and the repository is updated continuously during the annotation process

ODMedit is a tool to support uniform semantic annotation of patient data items. It is a semiautomatic approach, i.e., several codes from the portal are proposed and then a human expert decides about most appropriate coding. Data integration is simplified if the same code is applied for all data items with the same (or at least very similar) meaning. The high-level workflow is depicted in Fig. 2. The key idea is to re-use annotation codes from an expert-curated metadata repository to achieve uniform codes even when the terminology provides multiple coding variants. Several users of the system incrementally increase the number of curated annotation codes in the repository.

Initially, a search for items with similar names from the repository is conducted to annotate a new data item. A human expert determines whether the new data item has the same meaning as an existing data element. Data item context such as items from the same documentation form can be taken into account for this comparison: A human expert can review forms where the same item names were used. Based on this additional information he/she can take a decision about appropriate codes. Concepts can be overlapping or a concept can be a subclass of another (for example atrial fibrillation is a subclass of cardiac arrhythmia). Human experts can select codes according to the maximum specificity principle.

If no suitable annotation is available in the repository, matching annotation codes are retrieved from UMLS. If a single matching code is not available, postcoordination can be applied, i.e., combination of several codes. Definitions of UMLS codes are reviewed by human experts to identify matching codes. New data elements and their semantic annotation are added to the repository for future searches. Therefore annotation codes are available when the next data item with the same meaning shall be annotated. This enables uniform annotation of data items, even when several UMLS codes with similar meanings are available. The decision whether two data items have the same meaning is taken semi-automatically – i.e., computer-based suggestion with expert review – to ensure high coding quality.

### Evaluation

The scope of the evaluation is to demonstrate that this software tool is able to perform uniform semantic annotation for real data models from clinical studies. CDISC develops international data standards for clinical research. As a result of the Clinical Data Acquisition Standards Harmonization (CDASH) [18] initiative, CDISC developed a set of 22 forms with 292 frequently used data items, for instance regarding demographics data or adverse events. These items are coded with CDASH codes. It is determined how many of these data items can be annotated with UMLS codes. Correctness of UMLS codes is assessed by manual comparison with CDASH codes.

In addition, a set of 1,495 data models from the MDM portal was manually processed with ODMedit to determine technical feasibility of this tool.

ODMedit is intended to foster uniform semantic annotation. A random set of data elements from an established data standard was selected to test this feature. For each of those data elements available UMLS codes were identified with the UMLS Metathesaurus Browser [8]. Suitable codes were identified from the output of the UMLS Metathesaurus Browser by manual review. Available annotations in the MDM portal were analysed for each data element regarding uniform semantic annotation and compared to UMLS codes.

## Results

A reference implementation for uniform semantic annotation of patient data items was designed and implemented in R [19]. The web-based user interface was programmed with FastRWeb [20]. Queries to the repository were implemented with SQL commands to the MySQL database of the metadata repository [15]. Access to ODMedit is available at https://odmeditor.uni-muenster.de/. It is integrated into the metadata repository as an editor for semantic annotation of data items.

**Fig. 3:**
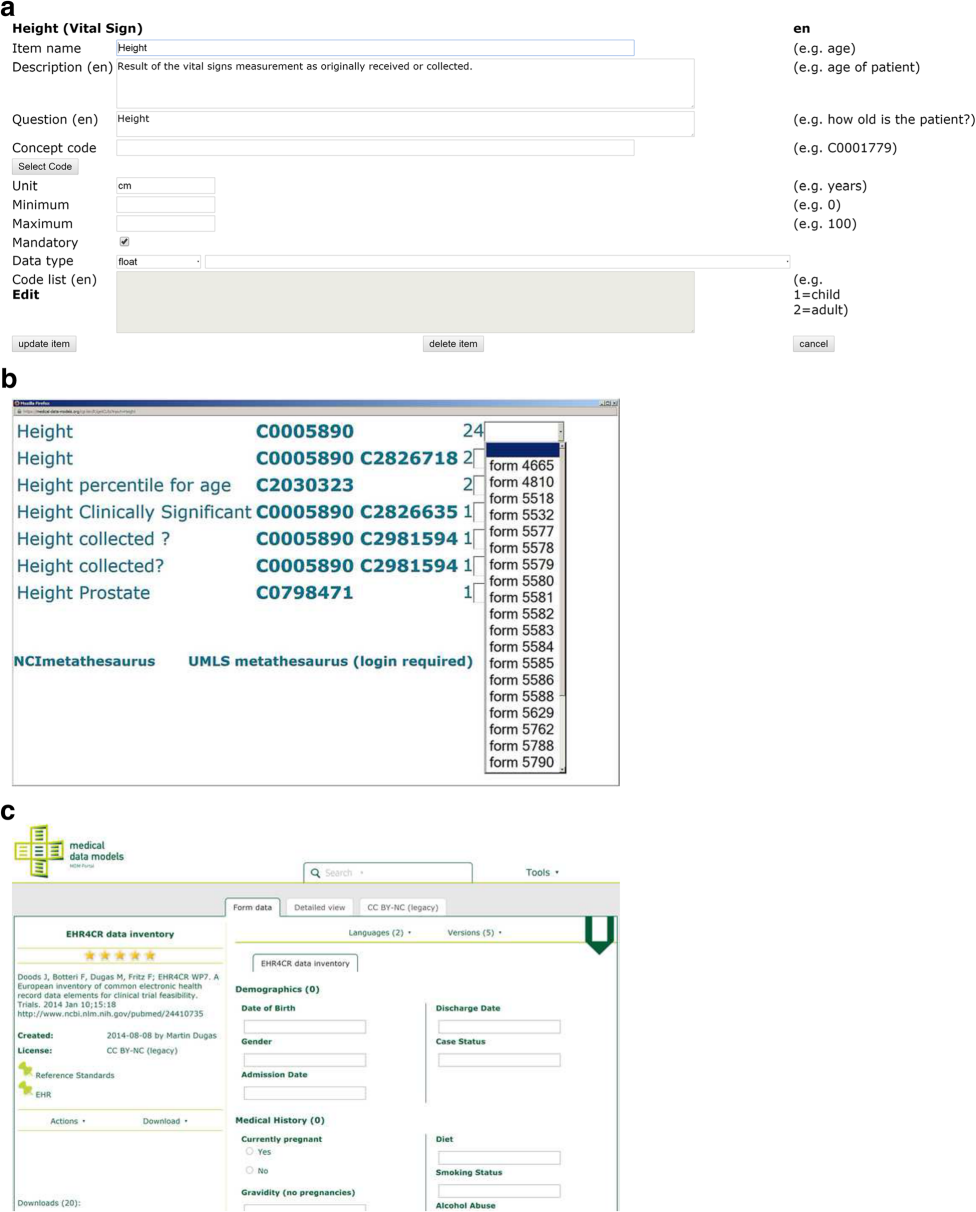
**a**: Summary of data item “height” within item group “Vital Sign”. **b**: Items with similar names like “Height” in the metadata repository. **c**: Form 5518 is presented, which is referenced by b. “Height” refers to “Body Height” in this example

Figure 3a presents a simple example for a data item. According to the ODM standard, it contains item name, description, question, minimum, maximum, data type and code list.

Figure 3b depicts how item annotations from the metadata repository can be re-used to code this data item: Several items with similar names like “Height” are already available in the repository. The number at the end of each line indicates how many times an item name/code combination was used. With the pulldown-menu on the right hand side more details about each item name/code combination are available, in particular related documentation forms. An expert can review the context of previously annotated items and decide whether a matching item is already available in the metadata repository. Figure 3c presents form 5518, which is referenced in Fig. 3b. “Height” (C0005890) refers to “Body Height”, therefore this code can be used in Fig. 3a. If no matching codes can be found within the portal, other semantic codes can be retrieved from UMLS metathesaurus. A query regarding “Height” in the UMLS metathesaurus browser shows 593 results (as of September 2015); therefore it is a non-trivial task to assign uniform codes even for relatively simple concepts like “Height”.

**Fig. 4:**
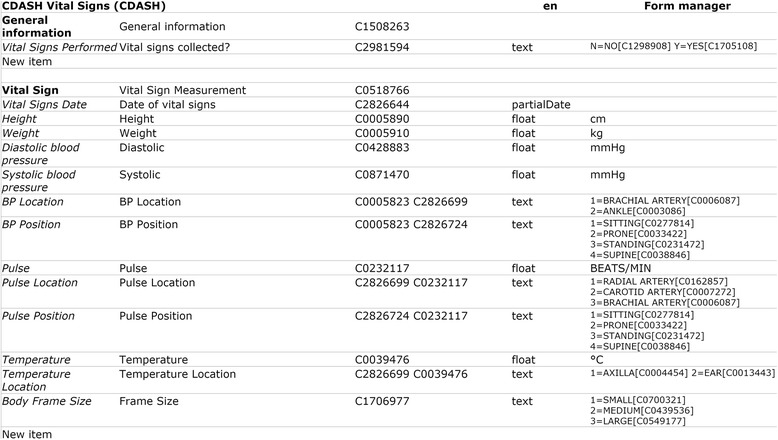
CDASH form “Vital Signs” with semantic annotations (UMLS codes) for all patient data items. Codelists, for example regarding Blood Pressure (BP) location, were also semantically annotated. Column one corresponds to UMLS terms, column two (similar, but not identical) to text labels on this form

ODMedit (as of April 2016) contains ~800,000 terms with semantic annotations which were derived from ~5,800 models in the portal of medical data models. This list of terms is updated automatically each time when a model is inserted or updated in the MDM portal. ODMedit was evaluated by annotation of CDASH forms. All 292 data items were annotated manually with ODMedit (without using code mappings of CDISC codes) and are available at https://medical-data-models.org/welcome/search?title=CDASH. Correctness of annotations was manually verified by comparison of UMLS concept definitions with CDASH terminology codes. As an example, Figure 4 presents CDASH form “Vital Signs”.

To assess technical feasibility, data models of the MDM portal were manually updated using ODMedit. In a six-week timeframe from May to July 2015, overall 1,495 data models were processed by seven users with ODMedit.

Five data elements were randomly selected from the EHR4CR data inventory [21]: Body weight, Date of Birth, Creatinine in Serum, Platelets and ALT. ODMedit intends to support uniform semantic annotation. Ideally, for each data element only one semantic annotation should be applied. Table 1 presents results from this analysis: For each data element there are between 9 and 23 matching UMLS codes. For one data element (body weight) uniform coding was achieved. For two data elements (Date of Birth, ALT) two coding variants were used, for another two data elements (Creatinine in Serum, Platelets) three variants.

**Table 1:** Semantic annotations of five randomly selected data elements in the MDM portal. For "Body weight" uniform annotation with C0005910 was achieved, for other data elements domain experts selected 2-3 coding variants

Data element	#UMLS codes	#Matching UMLS codes	#Occurences in MDM portal	Semantic annotation in MDM portal
Body weight	276	23	86	86x C0005910 Body weight
Date of Birth	55	9	85	55x C0421451 Patient date of birth 30x C0011008 Date in time C0027361 Persons C0005615 Birth
Creatinine in Serum	182	13	66	44x C0201976 Creatinine measurement, serum 13x C0010294 Creatinine 9x C0201975 Creatinine measurement
Platelets	249	16	229	213x C0005821 Blood Platelets 12x C0942474 Platelets:NCnc:Pt:Bld:Qn 4x C1287267 Finding of platelet count
ALT	104	13	37	30x C0201836 Alanine aminotransferase measurement 7x C0001899 Alanine Transaminase

## Discussion

Personalised medicine is data-intensive [22], but not only regarding genomic data. In contrast to genomic profiles, few attention has been given so far to the complexity and heterogeneity of patient data, the “phenotype”. An indicator for this complexity is the number of concepts in medical terminologies: >300,000 concepts in SNOMED CT, >2 million concepts in UMLS. The grand challenge of semantic interoperability between medical data sources is well-known for decades [23]. However, UMLS, SNOMED CT and many other medical terminologies – in contrast to classifications like ICD – don’t provide uniform coding, i.e., there can be several matching codes for a data element. To some extent, matching is subject to interpretation. For example, height can be coded in UMLS as C0489786 or C0005890. To foster data integration, uniform coding is highly desirable: I.e. the same code for “height” in any information system, when it is a candidate for data integration. For this purpose, it doesn’t matter whether code C0489786 or C0005890 is chosen, but it should be the same code in any system. For this reason ODMedit is connected to a large metadata repository and human experts can choose from a list of semantic annotations for potential re-use. Our evaluation demonstrates that several synonymous codes exist for a data element in UMLS (in our case between 9 and 23); therefore uniform annotation – the same code for the same meaning – is a challenging task. Uniform coding was achieved in one data element. Between two and three coding variants were identified for the remaining data elements, which is not yet perfect, but a lot better than a direct UMLS search with hundreds of potential hits. Future work shall address number and relevance of proposed concepts by ODMedit. In addition, interrater reliability of coding with ODMedit shall be assessed. It has to be taken into account that the final decision about coding is taken by human experts, because fully automated coding approaches have limitations [24]. More formal guidance how to assign uniform codes needs to be developed in the future, which is a lot of work given the amount of terms in UMLS.

The public discussion about patient data is dominated by data protection and privacy issues, which are absolutely important. Maybe as a side effect of this discussion, the vast majority of patient metadata – and implicitly their semantic annotation – is currently also kept secret [9]. However, this is a roadblock to semantic interoperability: It is simply impossible to integrate patient data between systems and share best practice in medical data structures if the available data items are kept secret.

The second important challenge for patient data integration is semantic annotation, because only data items with the same meaning shall be merged. The benefits of UMLS-based semantic annotation for data integration have been described previously [25]. Ideally, semantic annotation should be done in the very beginning by the author of each data item, because he or she is the one to know what exactly is meant by each data item. However, most patient data sources do not yet provide semantic codes. A first step is semantic annotation of metadata at a later stage with dedicated methods like ODMedit to facilitate data integration. Given the semantic richness of patient data requiring millions of codes, an international collaborative effort is needed to develop and maintain these annotations. UMLS was chosen, because it is composed from more than 100 major source vocabularies and therefore outperforms other terminologies regarding overall coverage of concepts. UMLS provides more than 4 million terms, but for some data elements like “Door-to-balloon time” [26] (regarding percutaneous coronary interventions) or “history of ibuprofen” an appropriate code is not yet available. Postcoordination, i.e., combination of several codes, helps to deal with semantic richness of patient data, but impedes uniform annotation, because different approaches how to perform postcoordination are available. The relationship between UMLS terms (UMLS Semantic Network) is currently not taken into account within ODMedit. There is an ongoing debate about the quality of hierarchical structures in major vocabularies from UMLS such as SNOMED CT: “The SNOMED CT hierarchies cannot be relied upon in their present state in our applications.” [27] The goal of uniform semantic annotation is to determine whether two data elements from different sources have the same meaning - yes or no. When two data elements have similar, but not identical meaning – as indicated by different semantic annotations –, review by domain experts is useful to assess whether data integration is feasible.

The context of a data element needs to be taken into account for appropriate semantic annotation. For example, an item “complication” can have a very different meaning in a controlled trial and an EHR system. ODMedit provides access to the complete documentation form for each annotation code, thereby enabling manual review of context.

### Related work

The proposed annotation tool ODMedit is based upon the CDISC ODM standard. There are several international resources available for data elements with semantic annotations, including cancer Data Standards Registry and Repository (caDSR) from NCI [28], OpenEHR Clinical Knowledge Manager [29] and Clinical Information Modeling Initiative (CIMI) [30]. However, these resources are currently not based on the ODM standard, which is recommended for metadata and data transfer by regulatory authorities for clinical research [12].

Mapping clinical data elements to controlled terminologies has been described in the literature. For instance, within the eMERGE network 157 data elements from 5 sites were mapped to caDSR, CDISC SDTM, NCI-T and SNOMED CT using a dedicated toolkit called eleMAP [31]. Another approach for mapping local data elements to standard vocabularies was proposed by German [32]. It is based on full text search in a dedicated ontology (like LOINC for laboratory values) combined with review by a local terminology expert. This publication clearly identifies the need for uniform semantic annotation: “The largest barrier to linking knowledge-based medical decision support systems to heterogeneous DBs is the variety of ways in which similar data are represented”. Our approach is based on a public metadata repository of annotated data elements. These annotations are re-used and only for new data elements an annotation code from UMLS is identified. Thereby uniform annotation is supported. Mapping eligibility criteria of clinical trials to semantic annotations is a complicated task, because many of these criteria are complex and sometimes ambiguous. In addition to NLP techniques manual processing is needed in many cases [33].

ODMedit is working on metadata only, not on data. For this reason it is a different approach than most semantic web approaches, addressing a web of linked data [34]. Approaches for mapping of ontologies to clinical databases have been described previously, for example ONTOFUSION [35]. ONTOFUSION applies automated unification of semantic codes. In contrast, ODMedit uses expert-based coding based upon a large-scale metadata repository of medical data models. According to our experience with >1,000 models, available medical terminologies are not yet in a development stage that allows fully automated and reliable semantic coding.

Many powerful tools to support patient data integration are already available, such as Internet technology to share metadata and connect information systems, metadata standards like CDISC ODM as well as sophisticated medical terminologies like SNOMED CT or UMLS to annotate data items. ODMedit demonstrates that uniform semantic annotation of patient data is a challenging task, but feasible in principle. This annotation can facilitate data integration [24]. However, adoption by the scientific community is needed to make an impact. As a first step, ODMedit is connected to the largest public portal of medical data models. ODMedit is limited to structural metadata. Therefore data-related aspects of data integration, for example data completeness, are not addressed by this tool.

Much more awareness is needed regarding the benefits of open metadata in medicine and beyond to overcome the currently existing silos of complex, non-standardised systems. Patient data is sensitive and needs to be de-identified appropriately before sharing – but structural metadata should be open and semantically annotated for the scientific community and all citizens.

## Conclusions

Semantic annotation of patient data structures is an important and yet unsolved grand challenge for medicine. Uniform manual semantic annotation of medical data models is feasible in principle, but requires a large-scale collaborative effort due to the semantic richness of patient data. A web-based tool for these annotations is available, which is linked to a public metadata repository.

## Abbreviations

caDSR: cancer data standards registry and repository
CDASH: clinical data acquisition standards harmonization
CDISC: Clinical Data Interchange Standards Consortium
CIMI: clinical information modeling initiative
CRF: case report form
EHR: electronic health record
EMA: European Medicines Agency
FDA: food and drug administration
ICD: international classification of diseases
MDM: medical data models
ODM: operational data model
SNOMED: systematized nomenclature of medicine
UMLS: unified medical language system
